# Porcine epidemic diarrhea virus strain CH/HLJ/18 isolated in China: characterization and phylogenetic analysis

**DOI:** 10.1186/s12985-023-02233-6

**Published:** 2024-01-24

**Authors:** Yuyao Guo, Ling Sui, Deming Kong, Dan Liu, Yueyi Gao, Yanping Jiang, Wen Cui, Jiaxuan Li, Yijing Li, Li Wang

**Affiliations:** 1 College of Veterinary Medicine, Northeast Agricultural University, Harbin, 150030 China; 2 Heilongjiang Key Laboratory for Animal Disease Control and Pharmaceutical Development, Harbin, 150030 China; 3 China Institute of Veterinary Drug Control, Beijing, 100081 China

**Keywords:** Porcine epidemic diarrhea virus, S protein, Isolation and identification, Phylogenetic evolution, Pathogenicity

## Abstract

**Background:**

Porcine epidemic diarrhea (PED) is an infectious disease of the digestive tract caused by the porcine epidemic diarrhea virus (PEDV), characterized by vomiting, severe diarrhea, and high mortality rates in piglets. In recent years, the distribution of this disease in China has remarkably increased, and its pathogenicity has also increased. PEDV has been identified as the main cause of viral diarrhea in piglets. This study aimed to understand the genetic evolution and diversity of PEDV to provide a theoretical basis for the development of new vaccines and the prevention and treatment of PED.

**Methods:**

A PEDV strain was isolated from the small intestine of a diarrheal piglet using Vero cells. The virus was identified using reverse transcription-polymerase chain reaction (RT-PCR), indirect immunofluorescence assay (IFA), and transmission electron microscopy. The whole genome sequence was sequenced, phylogenetic analysis was conducted using MEGA (version 7.0), and recombination analysis was performed using RDP4 and SimPlot. The S protein amino acid sequence was aligned using Cluster X (version 2.0), and the S protein was modeled using SWISS-MODEL to compare differences in structure and antigenicity. Finally, the piglets were inoculated with PEDV to evaluate its pathogenicity in newborn piglets.

**Result:**

PEDV strain CH/HLJ/18 was isolated. CH/HLJ/18 shared 89.4–99.2% homology with 52 reference strains of PEDV belonging to the GII-a subgroup. It was a recombinant strain of PEDV BJ-2011-1 and PEDV CH_hubei_2016 with a breakpoint located in ORF1b. Unique amino acid deletions and mutations were observed in the CH/HLJ/18 S protein. The piglets then developed severe watery diarrhea and died within 7 d of inoculation with CH/HLJ/18, suggesting that CH/HLJ/18 was highly pathogenic to newborn piglets.

**Conclusion:**

A highly pathogenic recombinant PEDV GII-a strain, CH/HLJ/18, was identified in China, with unique deletion and mutation of amino acids in the S protein that may lead to changes in protein structure and antigenicity. These results will be crucial for understanding the prevalence and variation of PEDV and for preventing and controlling PED.

**Supplementary Information:**

The online version contains supplementary material available at 10.1186/s12985-023-02233-6.

## Background

Porcine epidemic diarrhea (PED) is a highly infectious disease characterized by severe diarrhea, vomiting, and dehydration caused by the porcine epidemic diarrhea virus (PEDV) [1, 2]. PEDV can infect pigs of all ages, but piglets under two weeks old have a mortality rate as high as 100% [3]. In 1971, the disease was first reported in the United Kingdom and then spread to other European countries, such as France and Germany [1, 3, 4]. In 1978, Belgium took the lead in isolating the pathogen CV777 [1]. Since 2010, new variants of PEDV have emerged in China, causing endemic and periodic outbreaks of the disease [5, 6]. According to epidemiological investigations, 31 provinces in China reported positive PEDV cases in 2018 [7, 8]. Reports suggest that the outbreak of PED in China is the result of a new variant strain and that the original vaccine is unable to provide complete protection to the pig herd against PEDV [9]. Currently, PEDV is the main pathogen causing piglet diarrhea in pig farms, causing huge economic losses to the Chinese pig industry.

PEDV belongs to the Coronaviridae family of the order Nidovirales, a member of the genus Alphacoronavirus [10, 11]. PEDV is a single-stranded positive-sense RNA virus with a genome length of approximately 28000 nucleotides (nt), including a cap at the 5’ end, a Poly(A) tail at the 3’ end, and seven open reading fragments (ORFs; ORF1a, ORF1b, and ORF2-6) [3]. The polyproteins (pp1ab and pp1b) encoded by ORF 1a and 1b are cleaved by virus-encoded proteases into 16 non-structural proteins (nsp) [12]. ORFs 2–6 encode four structural proteins: spike glycoprotein (S), membrane protein (M), nucleocapsid protein (N), envelope protein (E), and the accessory protein ORF3 [12]. The S protein is the most important protein for virulence, and different PEDV strains frequently exhibit amino acid insertions, deletions, and mutations in their S proteins [13]. Therefore, the S protein is commonly used in epidemiological studies involving phylogenetic analysis and investigation of PEDV virulence [13–15]. ORF3 is an ion channel protein [16] used to distinguish between wild-type and attenuated PEDV strains, as attenuated vaccine strains have continuous deletions of 17 amino acids (positions 82–99) [17]. A large number of ORF3 gene-deleted viral strains have been reported in China, and the spontaneous deletion of ORF3 may be responsible for the decline in viral pathogenicity [18].

The PEDV is prone to variations in its replication process. Based on the phylogenetic analysis, the complete PEDV genome can be divided into two groups: GI (classic) and GII (variant) [19–21]. Both GI and GII have two subgroups: GI-a, GI-b, GII-a, and GII-b [19–21]. The GI-a subgroup mainly includes the prototype strain CV777 and its vaccine strain, as well as the classical Chinese strains. GI-b primarily comprises early or attenuated vaccine strains [22]. The GII genome contains epidemic strains that emerged in Asia at the end of 2010 and the current global epidemic strains, mainly in North America and Asia [22, 23]. Most GII-a subgroup strains originated in America, with a few originating from Asia, whereas GII-b subgroup strains are mostly endemic to Asia [23]. PEDV strains can also be divided into GI and GII based on whether the S gene contains an INDEL sequence [19, 23]. The GI group was further divided into GI-a (classical strains) and GI-b (S-INDEL strains) subgroups, based on the differences in the INDEL sequence. The GII group included GII-a (non-S-INDEL strains prevalent in Asia) and GII-b (non-S-INDEL strains prevalent in North America) subgroups [23, 24].

Recombination plays an important role in PEDV evolution [25]. In PEDV, recombination events occur in both the structural and non-structural protein-coding regions [25–27]. According to previous reports, PEDV SC-YB73 may be a recombinant of Chinese strains GDS47, TW/Yunlin550/2018, and Colombian COL/Cundiamarca/2014, with recombination event breakpoints located within the E, M, and N genes [25]. PEDV CH/HNQX-3/14 is a natural recombinant of attenuated vaccine strains (CV777 and DR13) and the Chinese strain ZMDZY, where potential recombination events may have occurred in the S, N, and ORF1a genes [26]. In addition, the highly pathogenic CN/Laoning25/2018 strain was created through the recombination of the low-pathogenicity PEDV strain SQ2014, which obtained the S gene of the highly pathogenic PEDV strain CN/GDZQ/2014, indicating that recombination of the S gene may alter the virulence of the virus [27]. Altogether, recombination increases the complexity of the genetic variants in PED, posing significant challenges to its prevention and treatment [28, 29].

To further understand the genetic evolution and diversity of PEDV, we isolated a novel PEDV strain (ch/hlj/18) belonging to the GII-a PEDV subgroup, elucidated its molecular characteristics, and determined its origin, evolution, and genetic relationships with reference strains. We assessed the pathogenicity of PEDV ch/hlj/18 in newborn piglets. Our findings revealed the genetic evolution and variant characterization of the prevalent strain of PEDV, which contribute to a better understanding of the epidemiology of PEDV and provide a scientific basis for the development of effective prevention and control strategies. Furthermore, the PEDV strain isolated in this study could be used as a candidate vaccine strain to develop a new vaccine against the pandemic strains in future studies.

## Materials and methods

### Cells, antibodies, animals, and clinical samples

The Vero cells (Number: CCL-81) were purchased from ATCC, and the mouse anti-PEDV N protein monoclonal antibody was made and donated by the Swine Disease Research Laboratory (School of Veterinary Medicine, China Agricultural University). The 3-day-old newborn piglets were purchased from the Experimental Animal Base of Harbin Veterinary Research Institute (Chinese Academy of Agricultural Sciences). Intestinal tissue samples were collected from piglets with diarrhea from a medium-scale pig farm in Heilongjiang, China. The pig farm had approximately 4000 sows, of which 98.1% of piglets in the delivery unit died within 3 d due to the outbreak of the PED epidemic.

### RNA extraction and RT-PCR analysis

The intestinal tissue samples were cut, ground in liquid nitrogen, and powdered. Every 0.5 mg of tissue powder was diluted with 1 mL of M199 (Gibco, NY, USA), thoroughly mixed, and centrifuged at 4500 × *g* at 4 ℃ for 5 min (Thermo Scientific Sorvall Legend Micro 17, Waltham, MA, USA). The supernatant was sterilized using a 0.22 μm filter (Biosharp, Hefei, China). Total RNA was extracted using the Fast200 Total RNA Extremely Fast Extraction Kit (Feijie, Shanghai, China) according to the manufacturer’s instructions. Reverse transcription was performed using a Superscript Reverse Transcriptase Reagent Kit (Takara, Tokyo, Japan). PEDV infection was evaluated using reverse transcription polymerase chain reaction (RT-PCR) with a specific primer pair (PEDV-N, Table 1).

### Virus isolation

Vero cells were cultured in T-75 flasks for PEDV replication. Vero cells were washed twice with phosphate buffer saline (PBS), inoculated with 2 mL of viral suspension, supplemented with 25 µg/mL trypsin (Gibco), and incubated at 37 °C in 5% CO_2_ for 1 h. The inoculum was removed, M199 containing 6 µg/mL of pancreatin was added, and the mixture was incubated at 37 °C for 48–72 h and propagated blindly for several passages until cytopathic effect (CPE) was observed. The final supernatants were collected, packed separately, and stored at − 80 °C. For purification of viruses, the supernatant was inoculated into Vero cells cultivated in 6-well plates according to the procedure mentioned above and incubated at 37 °C in 5% CO_2_ for 1 h. Following the removal of the inoculum, the cells in each well were overlaid with 0.5 mL of MEM (2×, Gibco), 0.5 mL of 0.8% low melting agarose, and 6 µg/mL trypsin. After spreading, the plates were placed upside down and incubated at 37 ℃ in 5% CO_2_ for 1 h. When the cells showed CPE after 48–72 h, the cells in each well were overlaid with 1 mL of covering solution (containing 0.1% toluidine blue), incubated at 37 ℃ in 5% CO_2_. When plaques were observed, a single plaque was placed into M199 medium and replicated in Vero cells.

#### IFA

Vero cells grown on 96-well plates were infected with PEDV for 24 h, and fixed with pre-cooled absolute ethanol at room temperature (20–25 ℃) for 30 min, and blocked with 0.3% bovine serum albumin (BSA) in PBS at 37 ℃ for 30 min. Then, the primary antibody, mouse anti-PEDV N protein monoclonal antibody was incubated at 37 °C for 2 h, followed by fluorescein isothiocyanate (FITC)-conjugated goat anti-mouse immunoglobulin G (IgG) (ZSGB-BIO, Beijing, China) at 37 °C for 30 min. The cells were counterstained with 4,6-diamidino-2-phenylindole (DAPI, Beyotime, Shanghai, China) at room temperature for 3 min. The fluorescence was visualized using an inverted fluorescence microscope (Leica, Wetzlar, Germany).

### Electron microscopy analysis

The supernatants of the PEDV-infected Vero cultures were centrifuged at 3000 × *g* for 30 min, and the precipitate was removed and recentrifuged at 13,000 × *g* for 30 min. The viral particles were resuspended in 100 µL of this supernatant, which was then negatively stained with 2% phosphotungstic acid (pH 7.0) and examined using transmission electron microscopy (Hitachi, Tokyo, Japan).

### Gene sequencing

Based on the PEDV genome sequences available in GenBank, 23 primer pairs were designed (Table 1). The whole genome was amplified by RT-PCR using the KOD-Plus Neo DNA polymerase (Toyobo, Tokyo, Japan) according to the manufacturer’s instructions. PCR products were sequenced by Comate Bioscience Co., Ltd. (Changchun, China). The verified genome sequence of the PEDV strain was submitted to GenBank.

**Table 1:** Primers used for whole genome amplification of PEDV CH/HLJ/18 using RT-PCR

Primer name		Sequence (5'–3')	Product size(bp)	
PEDV-1	PEDV-1-F	AAGTGCTGTGCTGTCCTCTAG	2032 bp	
	PEDV-1-R	TCTGTAAAACCCGTCACTCTC		
PEDV-2	PEDV-2-F	CTGCCTGTGACTGCTTAAAGG	2323 bp	
	PEDV-2-R	AACCATCAATAGCCATAGCAG		
PEDV-3	PEDV-3-F	GTCTGTGGTTGTGGTACTGG	1857 bp	
	PEDV-3-R	GTGGACAAAGACCATCCTCG		
PEDV-4	PEDV-4-F	GCTAGATTTAGGTTCAAGTCAG	2307 bp	
	PEDV-4-R	AACAGTAGGTATGGTAGTATGC		
PEDV-5	PEDV-5-F	ACGTGTATGCGTGTAGGTGC	2588 bp	
	PEDV-5-R	GTGAAACATAACCACTTGAAGAT		
PEDV-6	PEDV-6-F	TGGTAGCGACTTAGATGGTGTT	2440 bp	
	PEDV-6-R	GCAAAGTGTCAACAATGAAGAG		
PEDV-7	PEDV-7-F	ATCCTGCTAAGACCTACA	1949 bp	
	PEDV-7-R	GCTGTGTCATAGTGGGCA		
PEDV-8	PEDV-8-F	TAAGGATTTTGACTACTATAGGTATAATAGACCCACTG	631 bp	
	PEDV-8-R	AGCCAATTCATTGCACAACCTGAAAAAG		
PEDV-9	PEDV-9-F	GGGATTACCCAAAGTGCG	1968 bp	
	PEDV-9-R	CACGAGCGCGCTGTGGTAT		
PEDV-10	PEDV-10-F	GCTTAGTGCCCTATTACCAATTGATTGGTAAGC	626 bp	
	PEDV-10-R	AAAACACTGCTTGCTGTCAGGGTG		
PEDV-11	PEDV-11-F	CTCGTGTTGAGTGTTATGATGG	2479 bp	
	PEDV-11-R	TGGGTAGTGATGTCTTGTTTGT		
PEDV-12	PEDV-12-F	ACTCGCTGTAGGTCACCACT	2421 bp	
	PEDV-12-R	CTGCAGGCGCCTGAACATTA		
PEDV-S-1	PEDV-S-1-F	ATGAAGTCTTTAACCTACTT	1420 bp	
	PEDV-S-1-R	CCTGAGAACACTTGAGTTGG		
PEDV-S-2	PEDV-S-2-F	GCAGTAATTCCTCAGATCCTC	1164 bp	
	PEDV-S-2-R	GGTAGAAGAAACCAGGCAACT		
PEDV-S-3	PEDV-S-3-F	GTCACAATTAATTTCACTGGTCATGG	1325 bp	
	PEDV-S-3-R	GGTAGCTAACTGTAGAGCCTCTTC		
PEDV-S-4	PEDV-S-4-F	GATTCTGGACAGTTGTTAGC	2184 bp	
	PEDV-S-4-R	AGACTTTGAGACATCTTTGACA		
PEDV-ORF3	PEDV-ORF3-F	ACACTAGTTGACCTTGAGTGGC	1255 bp	
	PEDV-ORF3-R	GAACGCAGAGTACTTGTAATGGC		
PEDV-E	PEDV-E-F	TATCTAGCTATACGTGGGCGGC	617 bp	
	PEDV-E-R	AAAGACCCAATTGACCTGAAAG		
PEDV-M	PEDV-M-F	AGTCTTACATGCGAATTGACC	765 bp	
	PEDV-M-R	AGCTGACAGAAGCCATAAAGT		
PEDV-MN	PEDV-MN-F	GTCAATAGCATTCGGTTGTGGCG	841 bp	
	PEDV-MN-R	TTTGCACGTGAAGTAGGAGGTG		
PEDV-N	PEDV-N-F	CCGAGTTCGGTTCTCACAGAT	1415 bp	
	PEDV-N-R	CATAGCCTGAGGCATCAACAC		
PEDV-5’UTR	PEDV-5’UTR-R	GATTACGCCAAGCTTCTGGCACGATGTTACCACCACGACGAC		682 bp
PEDV-3’UTR	PEDV-3’UTR-F	GATTACGCCAAGCTTATTCCCAAGGGCGAAAATAGCGTAGC		871 bp

### Phylogenetic analysis and recombination

The ClustalW method using the DNA-STAR software was used to analyze multiple sequence alignments. Phylogenetic analysis was performed using the distance-based neighbor-joining method in MEGA (version 7.0). Bootstrap analysis was performed using 1000 replicates. The S protein amino acid sequence alignments were generated using ClustalX (version 2.0). PEDV strains used in this study are listed in Table 2.

**Table 2:** PEDV strains used in this study

Strain	ID	Country	Strain	ID	Country
15V010/BEL/2015	KR003452	Belgium	HUA-14PED96	KT941120	Vietnam
AH2012	KC210145	China	JS-A	MH748550	China
AH-M	KJ158152	China	KNU-1305	KJ662670	Korea
AJ1102	JX188454	China	KNU-1702	MH052681	Korea
BJ-2011-1	JN825712	China	KNU-1909	MN844888	Korea
CH/HBTS/2017	MH581489	China	LZC	EF185992	Korea
CH/HNQX-3/14	KR095279	China	NPL-PEDV/2013/P10	KJ778616	USA
CH/HNYF/14	KP890336	China	OH851	KJ399978	USA
CH/HNZZ47/2016	KX981440	China	OKY-1/JPN/2014	LC063847	Japan
CH_hubei_2016	KY928065	China	PEDV 1842/2016 ITA	KY111278	Italy
CH/JXJA/2017	MF375374	China	PEDV-7C	KM609204	China
CH/SCMY/2018	MH061343	China	PEDV-CHZ	KM609209	China
CH/SCZY44/2017	MH061338	China	PEDV-LS	KM609211	China
CH/SXWS/2018	MT090146	China	SD-M	JX560761	China
CH/ZMDZY/11	KC196276	China	SHXX1902	MN841671	China
CHM2013	KM887144	China	SM98	GU937797	Korea
CH/S	JN547228	China	USA/2014/IL/20697 P7	KT591944	USA
CV777	AF353511	Belgium	USA/Colorado/2013	KF272920	USA
DR13	JQ023161	Korea	USA/Indiana34/2013	KJ645641	USA
GDgh	MG983755	China	USA/lowa106/2013	KJ645695	USA
GD-1	JX647847	China	USA/Ohio69/2013	KJ645665	USA
GD-A	JX112709	China	USA/OK10240-8/2017	MG334555	USA
GD-B	JX088695	China	WHLL	MN037494	China
GER/L03209/2019	LR812926	Germany	YN1	KT021227	China
HLJBY	KP403802	China	YN90	KT021231	China
FR/001/2014	KR011756	France	ZJ15XS0101 P1	KX550281	China

Recombination analyses were performed using RDP4 (including RDP, GENECONV, Bootscan, MaxChi, Chimaera, SiScan, and 3Seq) and SimPlot to detect possible parental isolates and recombination breakpoints in PEDV CH/HLJ/18. The criteria for RDP4 to detect recombination and identify breakpoints were P < 10^− 6^ and a recombination fraction > 0.6.

### Prediction of the three-dimensional structure of the S protein

The amino acid sequence of the S protein was submitted to the SWISS-MODEL (https://swissmodel.expasy.org/) web server for predicting the three-dimensional protein model. The protein domains were colored, and the structural differences between the different S proteins were compared using PyMOL.

### Prediction of B-cell epitopes on the PEDV S protein

The continuous epitopes in the PEDV S protein were predicted using the Bepiprid 1.0 and 2.0 (http://tools.iedb.org/bcell/) tools in the online immune epitope database (IEDB) (https://www.iedb.org/). The threshold value of Bepippred 1.0 was set to 0.35, and that of Bepippred 2.0 was set to 0.5 [30]. DiscoTope (http://tools.iedb.org/discotope/) and ElliPro (http://tools.iedb.org/ellipro/) were used to predict discontinuous B-cell epitopes. ElliPro with a minimum score of 0.5. DiscoTope (version 1.1) used a threshold of − 7.7 [30].

### PEDV Infection in newborn piglets

Eight newborn piglets were randomly allocated to two groups: a sham-inoculated control group (*n* = 4) and a PEDV-inoculated group (*n* = 4). Each inoculated group was housed separately. The piglets were fed a commercial milk replacer (eight times daily). Piglets in group 1 were separately infected with 5 mL (5.1 × 10^3^ PFU/mL) of PEDV CH/HLJ/18 strains. Piglets in group 2 served as uninfected controls and were fed 5 mL of M199. Following inoculation, fecal morphology, degree of diarrhea, dehydration, and mental state of the piglets were monitored. All piglets were euthanized 7 d post-challenge.

### Histopathology and immunohistochemistry (IHC)

The small intestine tissue of the piglets was fixed in 4% paraformaldehyde for 48 h, and slices with a thickness of about 3 μm were prepared. The slices were then immersed twice in xylene for 10 min each. Absolute ethanol was used for the dehydration. Intestinal tissue samples were routinely stained with hematoxylin and eosin (beyotime, Shanghai, China) for histopathological analyses. For IHC, after washing in absolute ethanol, the slices were blocked with 3% H_2_O_2_ for 10 min at 25 °C, followed by the addition of 0.01 M sodium citrate buffer solution (pH 6.0). The membranes were blocked with 3% BSA for 20 min at room temperature. This was followed by incubation with a mouse anti-PEDV N protein monoclonal antibody and goat anti-mouse IgG (HRP) (Thermo Fisher Scientific) secondary antibodies for 1 h each. After staining with diaminobenzidine (DAB) and counterstaining with hematoxylin, slides were mounted and observed under a fluorescence microscope (Bio-Rad, Hercules, CA, USA).

## Results

### Successful isolation of PEDV CH/HLJ/18 strain

**Fig. 1:**
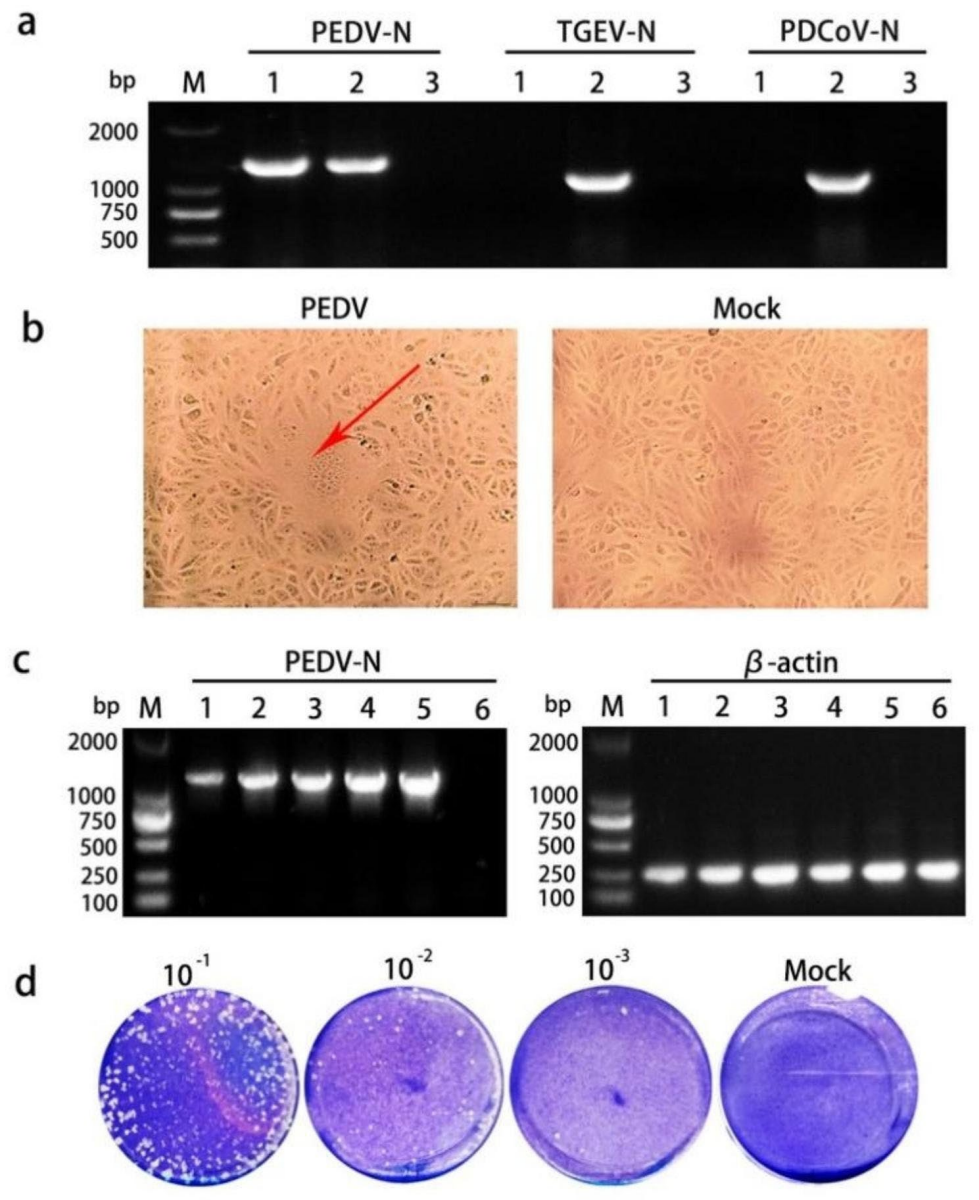
Isolation and cultivation of PEDV in Vero cells. (**a**) Identification of PEDV and absence of TGEV/PDCoV in small intestinal tissue sample. Lane 1: Small intestine tissue sample of diarrhea piglets; Lane 2: small intestine tissue sample of piglets infected with PEDV/TGEV/PDCoV as a positive control; Lane 3: non-template control (NTC). (**b**) Appearance of PEDV-infected and non-infected Vero cells in M199. (**c**) Identification of PEDV in Vero cells during serial passages using RT-PCR. Lanes 1–5: blind transmission of PEDV cell culture from the 1st to 5th generation; Lane 6: NTC. (**d**) Plaque morphology of PEDV at different dilutions using the direct agarose overlay plaque assay

Diarrhea-related viruses were detected in diarrheal piglet samples using RT-PCR, and only PEDV was detected in the sample, not the transmissible gastroenteritis virus of swine (TGEV) or porcine deltacoronavirus (PDCoV) (Fig. 1a). The supernatants of PEDV-positive samples were inoculated into Vero cells, and an obvious and typical CPE of PEDV was observed after five generations of blind transmission. Compared with uninfected Vero cells, PEDV-infected Vero cells were characterized by syncytial formation, cell rupture, and abscission (Fig. 1b). The RT-PCR results confirmed successful transmission of PEDV during serial passages (Fig. 1c). In the direct agarose overlay plaque assay, PEDV-CH/HLJ/18 formed plaques (Fig. 1d). Single plaques were selected, cultured, and used for purification and expansion. The virus titer was determined to be 5.1 × 10^3^ PFU/mL.

### Identification of isolated strains

**Fig. 2:**
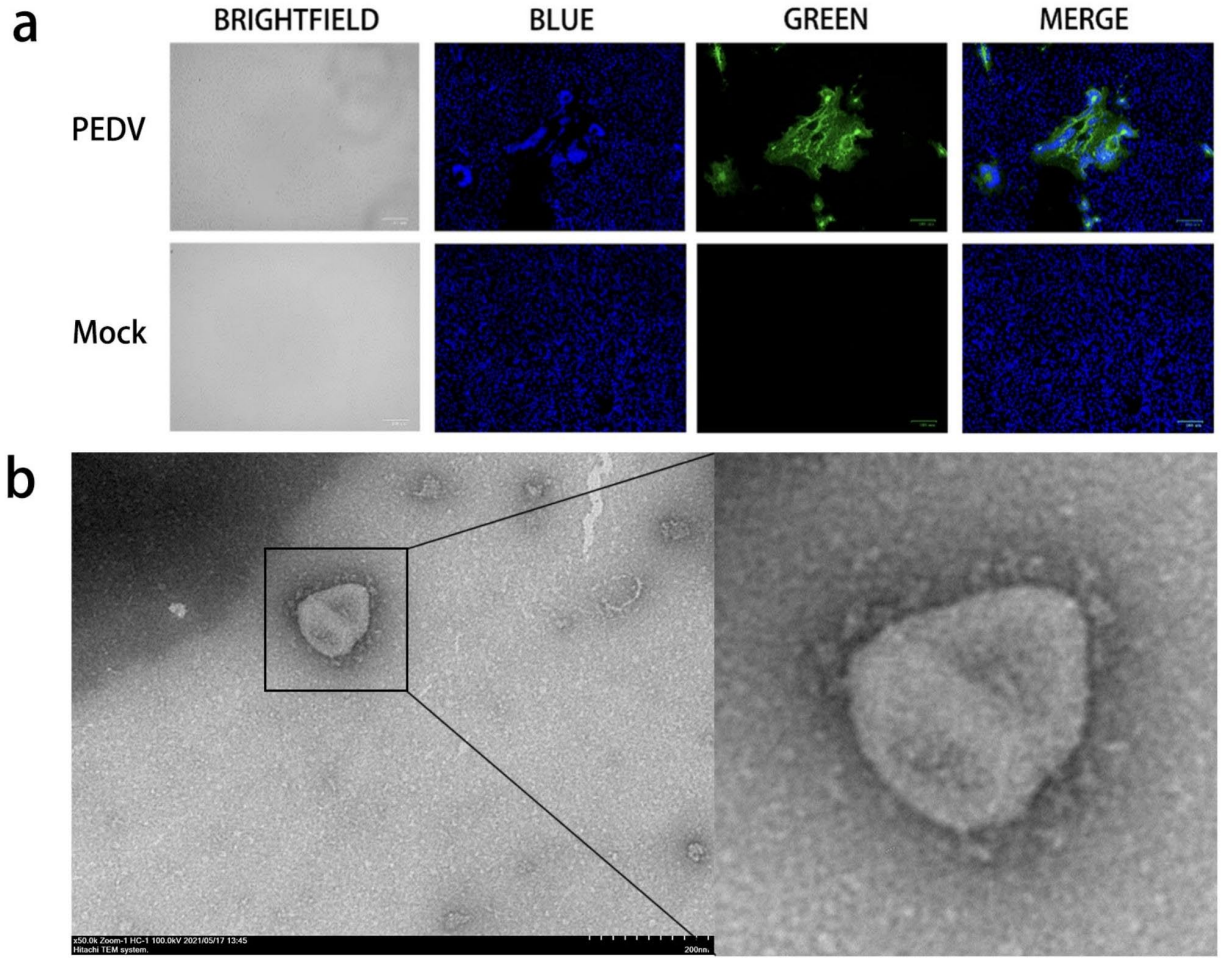
Detection of PEDV strain. (**a**) Immunofluorescence assay (IFA) of Vero cells infected with PEDV isolate. PEDV N was green (fluorescein isothiocyanate, FITC), and 4,6-diamidino-2-phenylindole (DAPI) was blue. (**b**) Electron microscopy images of PEDV strain CH/HLJ/18 particles. Bar = 200 nm. Magnification, ×50,000

The RT-PCR assay revealed that the sample was positive for PEDV (Fig. 1a). Sanger sequencing confirmed that the virus was PEDV. Vero cells infected with CH/HLJ/18 displayed a CPE typical of PEDV infection, and IFA revealed that CH/HLJ/18 specifically bound to the PEDV N protein antibody (Fig. 2a). Under the electron microscope, the viral particles appeared spherical with a diameter of approximately 100 nm and a coronal surface (Fig. 2b). These results confirmed that the isolate was PEDV. Hereafter, it is referred to as CH/HLJ/18.

### PEDV strain CH/HLJ/18: a member of the GII-a subgroup

**Fig. 3:**
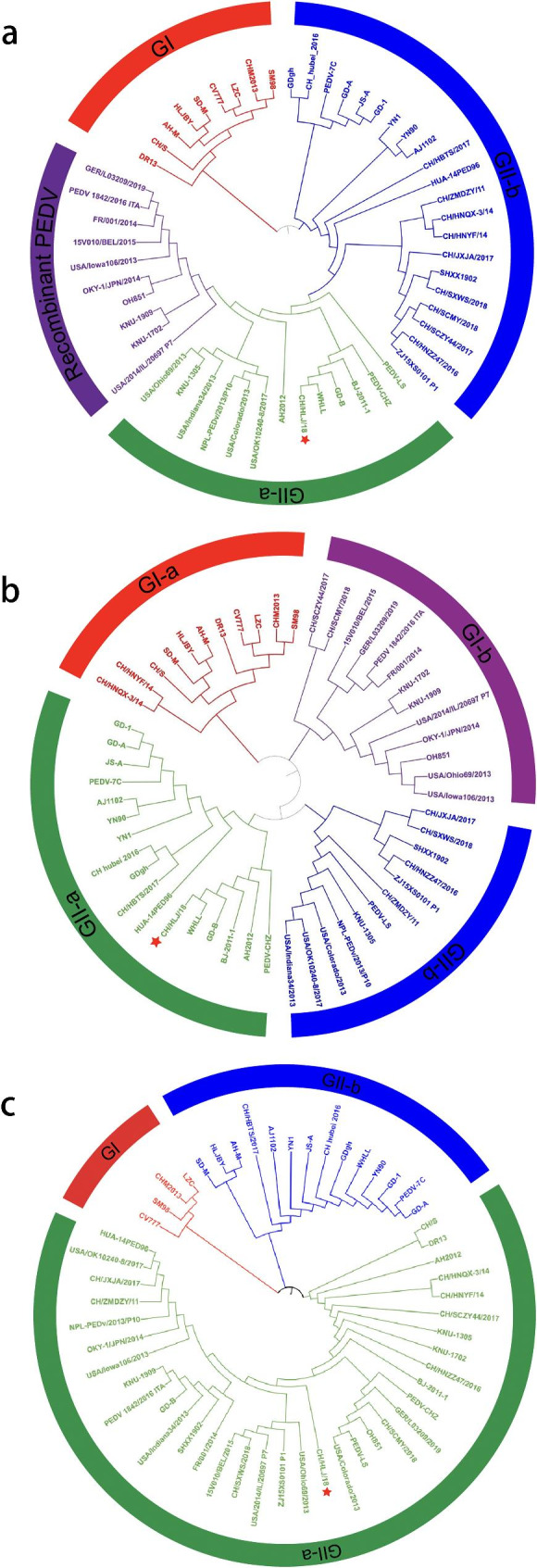
Phylogenetic analyses of PEDV available in GenBank. The tree was constructed using the neighbor-joining method (bootstrap n = 1000) based on MEGA7. (**a**) The whole genome sequence. (**b**) The S gene sequence. (**c**) The ORF3 gene sequence

The whole genome sequence of PEDV strain CH/HLJ/18 obtained by gene sequencing was 28,033 nucleotides (nt) long and was submitted to GenBank (accession number: MW561264). Based on the genome-wide sequence alignment, the nucleotide sequence homology between CH/HLJ/18 and the 52 representative strains ranged from 89.4 to 99.2%. The PEDV strain USA/OK10240-8/2017 showed the highest similarity (99.2% nucleotide identity) to CH//HLJ/18, and SM98 had the lowest similarity (89.4% nucleotide identity). Phylogenetic analysis based on the whole genome of PEDV reference strains indicated that CH/HLJ/18, belonging to the GII-a subgroup, was closely related to WHLL, GD-B, BJ-2011-1, and PEDV-CHZ, and was distinct from the classical strain CV777 (Fig. 3a). Based on the phylogenetic analysis of the S gene of the PEDV strain in GenBank, the genetic correlation between PEDV isolates was studied. Phylogenetic analysis of the S gene revealed that PEDV strains could be divided into three groups. The S gene of CH/HLJ/18 belonged to the GII-a group and was closely related to those in Chinese strains such as WHLL, GD-B, and BJ2011-1 (Fig. 3b). The nucleotide sequence homology of the S gene ranged from 91.6 to 99.6%. The nucleotide sequence homology of ORF3 ranged from 87.0 to 99.4%. These results confirmed that CH/HLJ/18 belonged to the GII-a group (Fig. 3c).

### Recombination within the PEDV CH/HLJ/18 ORF1b gene

**Fig. 4:**
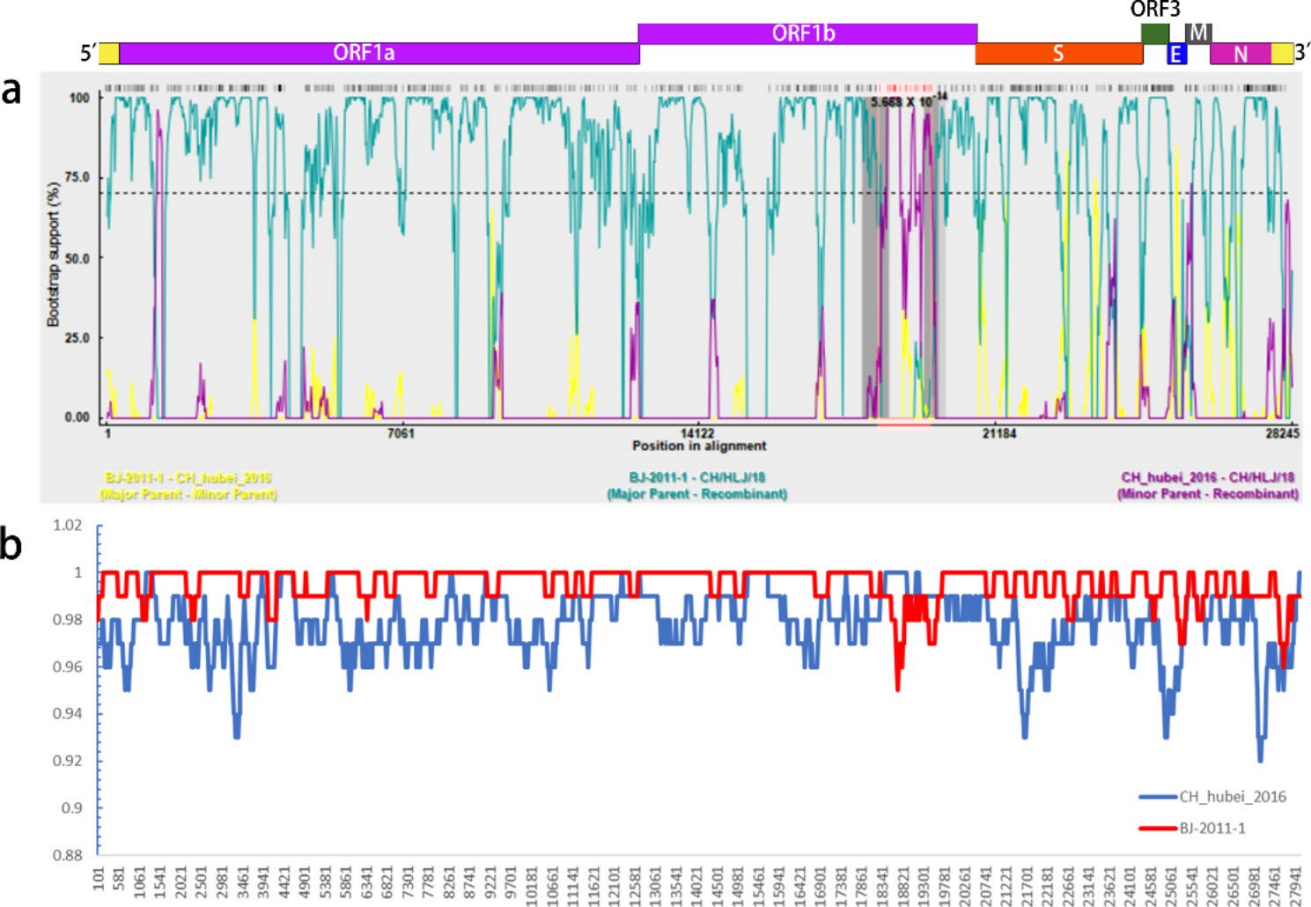
Cross-over regions in the genomes of BJ-2011-1 and CH_hubei_2016 identified by RDP4 and SimPlot. (**a**) RDP4 (Bootscan) showed that the recombination breakpoint was at ORF1b (nt18360). RPD4 was performed using default settings. (**b**) The SimPlot intersection area was consistent with the RPD4 results. The operating parameters used were the Kimura (2-parameter) distance model, 2.0 Ts/Tv ratio, neighbor-joining tree model, and 1000 Bootstrap replicates

To identify whether recombination events occurred in the PEDV CH/HLJ/18 strain, the constructed genome-wide phylogenetic tree was analyzed using RDP4 and SimPlot. Recombination analysis of RDP4 revealed a potential ORF1b recombination event in the CH/HLJ/18 strains, with a recombination score of 0.614 (Fig. 4a; Table 3). The breakpoint of the potential recombination region was located at nt 18,360–19,622 of the complete genome at the end of ORF1b. The major parental strain was BJ-2011-1 and the minor parental strain was CH_hubei_2016 (Fig. 4a; Table 3). This result was further confirmed by SimPlot analysis (Fig. 4b).

**Table 3:** Recombination events identified by recombination detection program 4 (RDP4).

Breakpoint position in recombinant sequence	Parental sequence				Av.P-Val for the six detection methods in RDP					
Recombinant segment	Begin	End	Major	Minor	GENECONV	BootScan	MaxChi	Chimaera	SiSCAN	3Seq
ORF1b	18,360	19,622	BJ-2011-1	CH_hubei_2016	1.730 × 10^− 06^	9.584 × 10^− 14^	1.577 × 10^− 04^	9.879 × 10^− 03^	8.567 × 10^− 03^	6.042 × 10^− 09^

### Deletions and mutations affect the antigenticity of the CH/HLJ/18 S protein

**Fig. 5:**
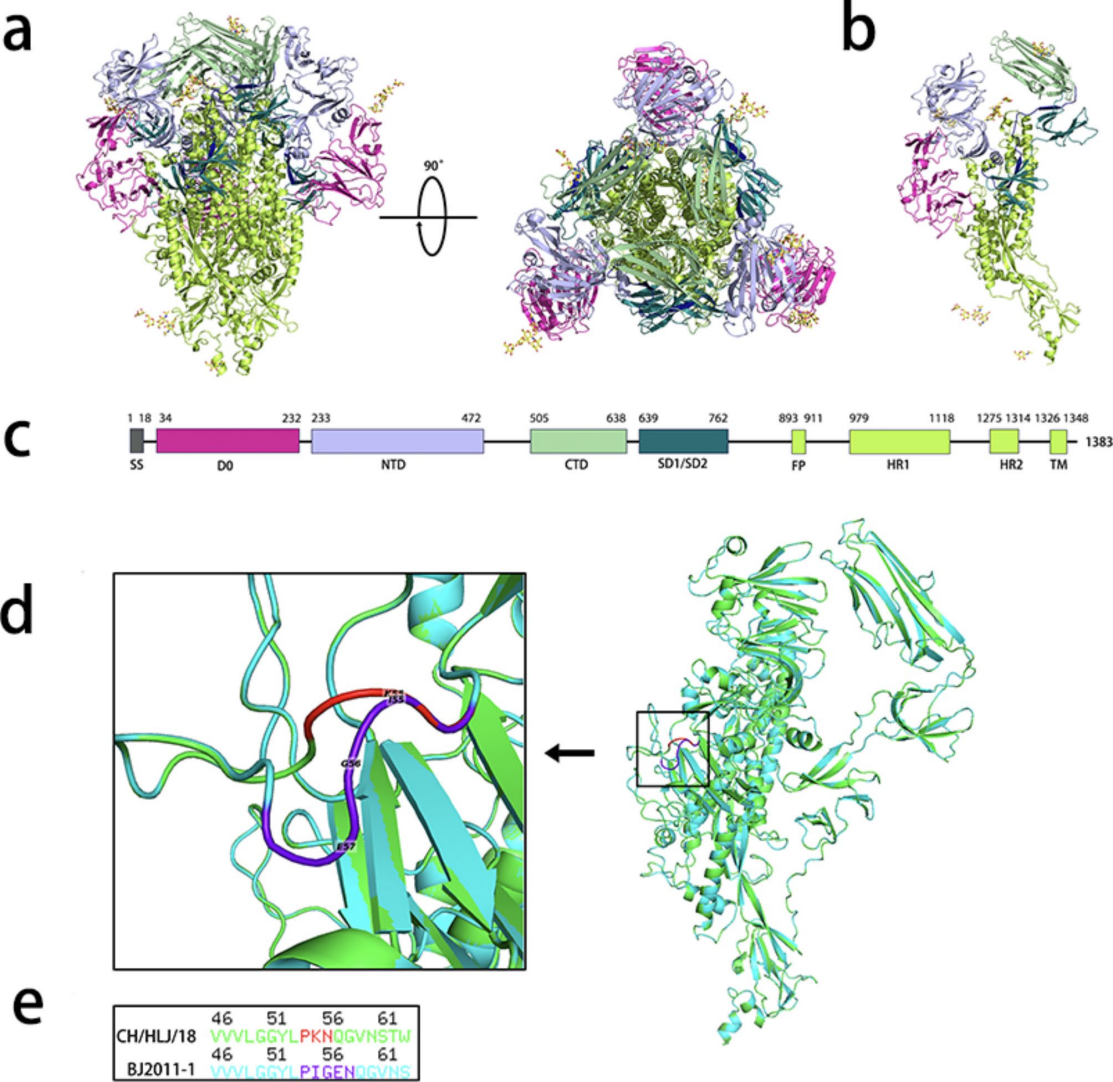
Mutation and deletion of amino acids cause structural changes in the PEDV CH/HLJ/18 S protein. (**a**) The predicted structure of the CH/HLJ/18 S protein trimer, with different colors representing different monomers. (**b**) The predicted structure of the S protein monomer. (**c**) Schematic representation of the S protein organization. Different colored boxes represent different domains of the S protein. Amino acids 473–504 are displayed in blue. (**d**) Comparison of predicted structures of CH/HLJ/18 and BJ2011-1 S monomers. The monomer structure of CH/HLJ/18 S protein is displayed in green, and the portion that differs from that of BJ2011-1 S protein is displayed in red. The monomer structure of BJ2011-1 S protein is cyan, and the part that differs from that of CH/HLJ/18 S protein is represented in purple. (**e**) Comparison of amino acid sequences of the structural differences between the S proteins of CH/HLJ/18 and BJ2011-1.

We compared the amino acid sequences and studied the genetic variation in the S protein of PEDV strains. Compared with the reference strains, CH/HLJ/18 showed 3 amino acid deletions (positions 57, 58, and 1389) and 3 unique amino acid mutations (positions 56, 71, and 1316) (Figure S1a). The tertiary structure of the S protein was predicted to study the effects of deletions and mutations on the structural changes in the CH/HLJ/18 S protein. The CH/HLJ/18 S protein contains N-terminal S1 and C-terminal S2 domains. S1 can be further divided into N-terminal domain (NTD), C-terminal domain (CTD), subdomain 1 (SD1), and subdomain 2 (SD2). The S2 domain includes the fusion peptide (FP), heptapeptide repeat 1 (HR1), heptapeptide repeat 2 (HR2), and transmembrane fragment (TM) (Fig. 5a-c). This finding is consistent with those of previous studies on the PEDV domain [31]. Unique amino acid deletions and mutations in the CH/HLJ/18 S protein are concentrated in the D0 domain. To compare the structural differences caused by unique deletions and mutations in the S protein, we selected the BJ2011-1 strain, as it is closely related to CH/HLJ/18 and belongs to the GII subgroup. The amino acid mutation at the 55th position of CH/HLJ/18 and deletion at the 56–57 positions led to a change in the protein structure (Fig. 5d-e). No structural changes were observed at other locations. Analysis of the continuous and discontinuous B-cell epitopes of the CH/HLJ/18 and BJ-2011-1 S proteins showed that the unique deletion (positions 57 and 58) and mutation (positions 56 and 71) of CH/HLJ/18 caused changes in the linear B-cell epitope (Table S1). Deletion (positions 57 and 58) and mutation (position 56) of CH/HLJ/18 also caused changes in the conformational epitopes (Tables S2-3). These results indicated that deletions and mutations lead to antigenicity changes in the CH/HLJ/18 S protein.

### Pathogenicity of PEDV strain CH/HLJ/18 in piglets

**Fig. 6:**
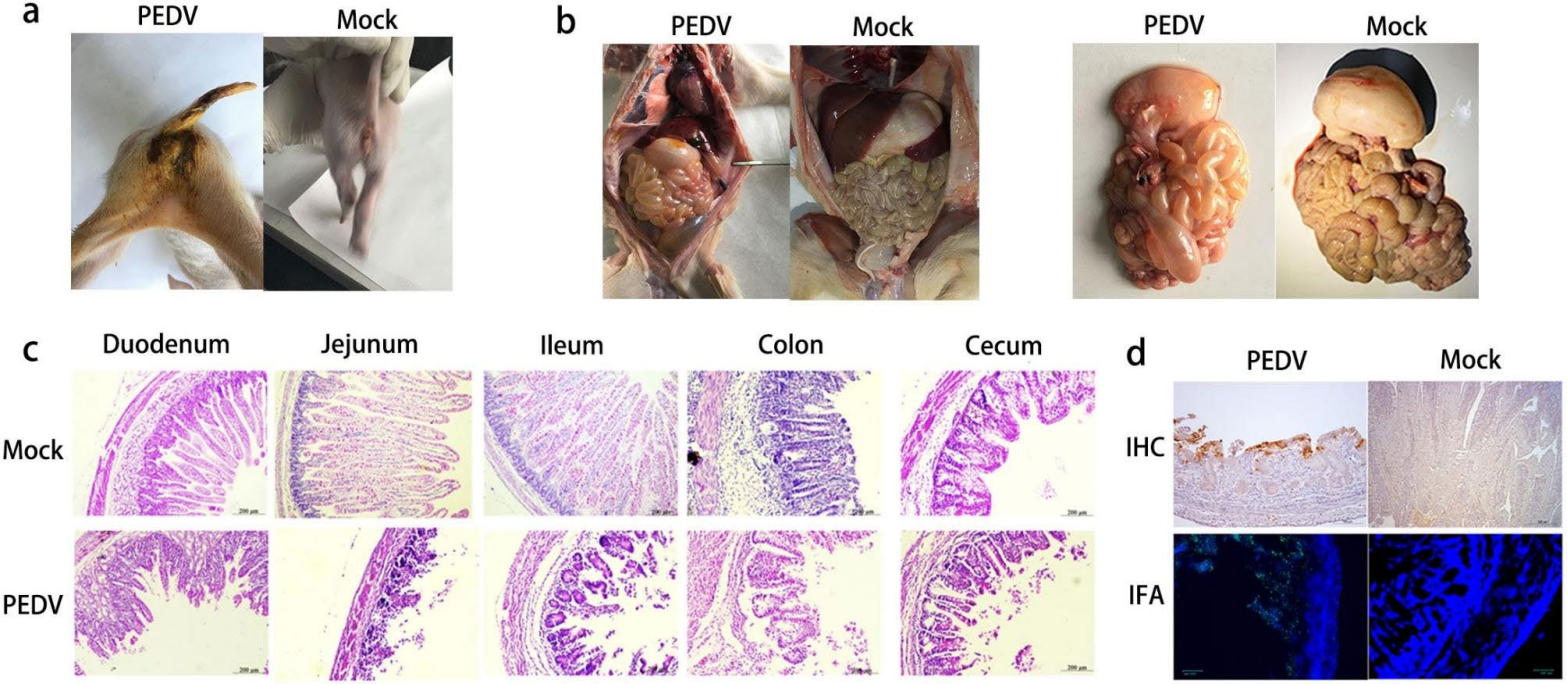
Macroscopic and microscopic intestinal lesions in piglets inoculated with the CH/HLJ/18 strain. (**a**) Clinical symptoms of piglets. (**b**) Intestines from representative piglets inoculated with CH/HLJ/18 and a negative control piglet were examined for gross lesions. (**c**) Histopathological examinations of the intestine of piglets inoculated with PEDV strain CH/HLJ/18 and control medium. (**d**) Microscopic small intestine lesions in piglets inoculated with the PEDV CH/HLJ/18 strain

The pathogenicity of CH/HLJ/18 was evaluated by experimentally infecting newborn piglets. At 24 h after challenge, all piglets in the infected group exhibited typical clinical signs of PEDV infection, including severe watery diarrhea, vomiting, and weakness. The stool around the perianal region was yellow, watery, and foul-smelling. No clinical symptoms were observed in the NC piglets (Fig. 6a). All piglets in the infected group died within 7 d of inoculation with PEDV. Anatomical observations showed that piglets in the infected group exhibited typical PED lesions in the intestine, including gastric distention, thinning of the intestinal wall, and watery intestinal contents (Fig. 6b). Histopathological examination of the intestine revealed typical features of viral enteritis, including shortening, shrinking, and even shedding of villi in the small intestine, with the most serious injuries occurring in the jejunum and ileum (Fig. 6c). Villus damage also occurred in the large intestine but was less severe than that in the small intestine (Fig. 6c). The PEDV antigen was detected in the small intestine after inoculation with the CH/HLJ/18 strain, resulting in a high concentration in severely atrophic small intestinal villi (Fig. 6d).

## Discussion

In recent years, the prevalence, range, incidence rate, and mortality due to PED have increased significantly in pig farms worldwide, causing huge economic losses to the global pig industry [3]. Since 2010, the identification and sequencing of PEDV strains have indicated that PEDV GII strains are highly prevalent in China [7, 22, 32]. In this study, we isolated a PEDV strain from fecal samples of piglets suspected of having viral diarrhea from pig farms in Heilongjiang Province, China. According to previous studies, PEDV has the highest detection rate among porcine viral diarrheal viruses in China [33, 34]. Our findings further demonstrate that PEDV is the main cause of viral diarrhea in piglets. The PEDV genome is divided into two groups: GI (GI-a and GI-b) and GII (GII-a and GII-b). CH/HLJ/18 belongs to the GII-a subgroup, which grows rapidly and is the dominant strain [33]. The shapes of the phylogenetic trees based on the S gene and entire genome were similar, whereas those based on other genes did not exhibit this similarity (Figure S2), indicating that the genetic evolution of these strains was mainly caused by variations in the S gene.

Recombination has played an important role in the genetic evolution of PEDV [35], increasing the genetic diversity of PEDV, making the virus more complex and heterogeneous, and posing a major challenge to the development of vaccines [3, 19, 35]. We identified that CH/HLJ/18 may be a recombinant strain derived from a GII-a strain (BJ2011-1) and GII-b strain (CH_hubei_2016), with a breakpoint in ORF1b. ORF1b is an important non-structural protein-coding region in the PEDV genome and is involved in the proliferation and synthesis processes of the virus [36]. Recombination events in ORF1b may result in variations in the viral genome that can affect viral viability and transmissibility. Our study identified the recombinants generated between different subgroups of PEDV strains in China. According to previous studies, the genome sequences of PEDV strains from China show the most recombination events, and the spread of PEDV may be an important reason for PEDV recombination [37]. The PEDV CH/HLJ/18 strain from Heilongjiang Province is the result of the recombination of PEDV strains from Hubei and Beijing. The emergence of the recombinant CH/HLJ/18 strain in Heilongjiang may be related to cross-provincial transportation of pigs [5, 37]. PEDV can be transmitted indirectly through contact with contaminated feed and transportation vehicles; therefore, transportation may play a critical role in PEDV transmission [38, 39]. Therefore, pig transport may be an important factor in the recombination of PEDV strains [19, 40]. This suggests that we should strengthen the management of pig breeding and environmental hygiene during transportation, improve management policies for cross-provincial transportation of pigs, and reduce the possibility of PEDV transmission and mutation.

The S protein is located on the surface of PEDV and is the main epitope neutralizing antibodies [41]. Variations in the PEDV S protein are the reason for the change in viral antigenicity, which reduces the effectiveness of traditional vaccines [42]. We analyzed the amino acid sequence of the CH/HLJ/18 S protein and found that it exhibited three unique amino acid deletions and three unique amino acid mutations. A comparison of 3D models of the S protein revealed that amino acid deletions and mutations in the D0 domain lead to changes in the structure of the S protein. We predicted that the mutated region might contain both continuous and discontinuous epitopes, indicating that the antigenicity of the virus might have changed. The emergence of new mutations may explain the failure of traditional vaccines, posing greater challenges to vaccine protection [43, 44]. CH/HLJ/18 shares high pathogenicity with its parent strain, indicating that changes in S protein antigenicity are not necessarily related to pathogenicity. Developing effective prevention strategies against highly pathogenic GII strains is urgently required for controlling PED epidemics in China. The data provided in this study will help develop a new type of vaccine against the PEDV strain prevalent in China.

## Conclusion

We identified a PEDV GII-a strain, CH/HLJ/18, that is highly prevalent in China. Our research showed that CH/HLJ/18 is a natural recombinant product of BJ2011-1 and CH_hubei_2016, and that the unique deletion and mutation of amino acids in the S protein led to changes in protein structure and antigenicity. In addition, CH/HLJ/18 had high pathogenicity in piglets, comparable to that of its parent strains. Our research provides a reference for understanding the epidemiology and genetic evolution of PEDV, and is of great significance in the prevention and control of PED.

### Electronic supplementary material

Below is the link to the electronic supplementary material.

Supplementary Material 1

Supplementary Material 2

## Data Availability

All data generated or analyzed in this study are included in the published article [and its supplementary information files].

## Abbreviations

PED: Porcine epidemic diarrhea
PEDV: Porcine epidemic diarrhea virus
S: Spike glycoprotein
E: Envelope protein
M: Membrane protein
N: Nucleocapsid protein
ORF: Open reading fragments
IHC: Immunohistochemistry
IFA: Immunofluorescence assay
RT-PCR: Reverse transcription-polymerase chain reaction
TGEV: Transmissible gastroenteritis virus of swine
PDCoV: Porcine deltacoronavirus
MEGA: Molecular Evolutionary Genetics Analysis
RDP: Recombination detection program
BSA: Bovine serum albumin
DAB: Diaminobenzidine
FITC: Fluorescein isothiocyanate
CPE: Cytopathic effect
NTD: N-terminal domain
CTD: C-terminal domain
SD: Subdomain
FP: Fusion peptide
HR: Heptapeptide repeat
TM: Transmembrane fragment
