# Supplementary material for: Porcine epidemic diarrhea virus strain CH/HLJ/18 isolated in China: characterization and phylogenetic analysis
Source: Virol J. 2024 Jan 24;21:28. doi: 10.1186/s12985-023-02233-6 (PMC10807084; doi:10.1186/s12985-023-02233-6)
Supplement: Supplementary file 1 — Supplementary Material 1 [file 12985_2023_2233_MOESM1_ESM.docx]

Porcine Epidemic Diarrhea Virus Strain CH/HLJ/18 isolated in China: Characterization and phylogenetic analysis

Yuyao Guo^1, 2^, Ling Sui^1, 2^, Deming Kong^1, 2^, Dan Liu^3^, Yueyi Gao^3^, Yanping Jiang^1, 2^, Wen Cui^1, 2^, Jiaxuan Li, Yijing Li^1, 2^, Li Wang^1, 2^*.

1 College of Veterinary Medicine, Northeast Agricultural University, Harbin 150030, China

2 Heilongjiang Key Laboratory for Animal Disease Control and Pharmaceutical Development, Harbin 150030, China

3 China Institute of Veterinary Drug Control, Beijing 100081, China

*Corresponding author: wanglicau@163.com (L.W.); Tel: +86-451-55190385 (L.W.)

**SUPPLEMENTARY INFORMATION**


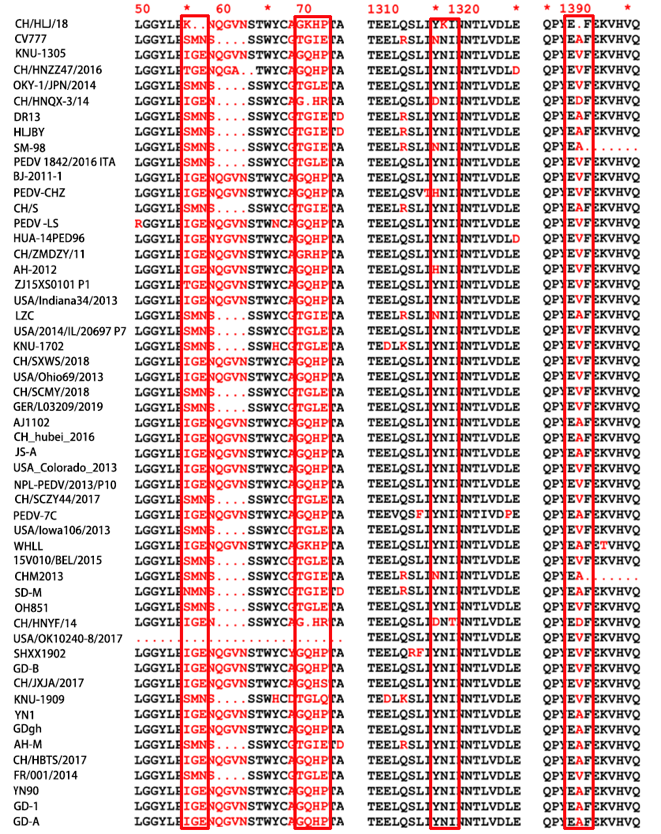


Figure S1. Amino acid sequences of the S protein of PEDV CH/HLJ/18 compared with those of the reference strains.


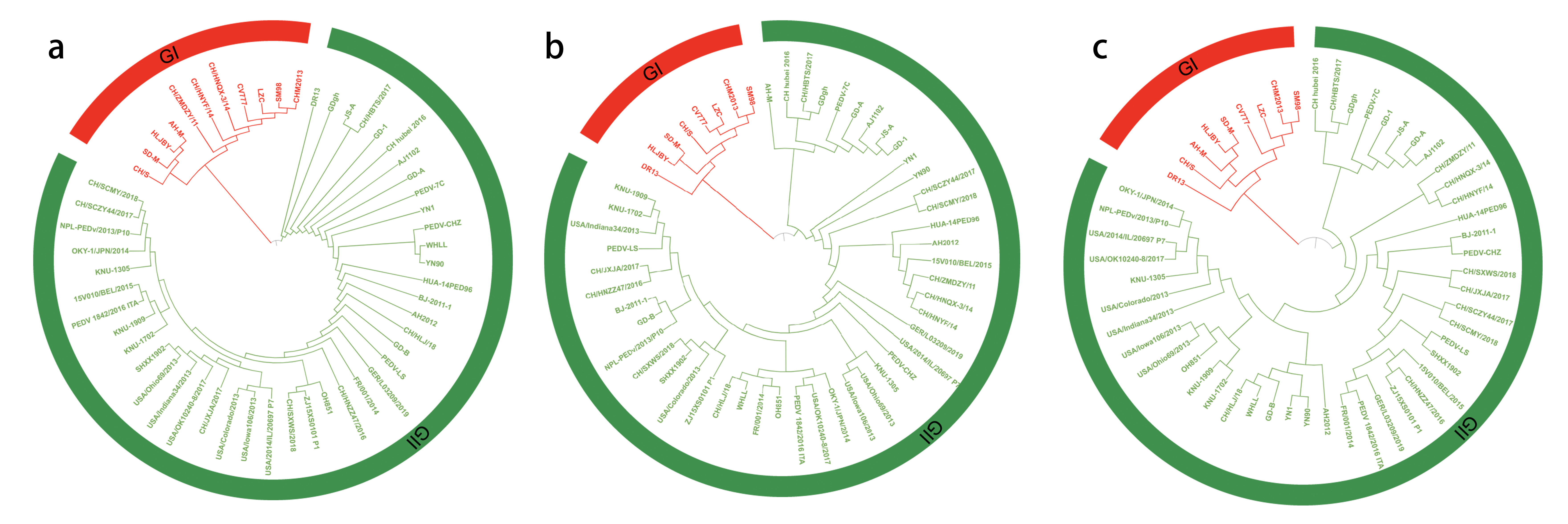


Figure S2. Phylogenetic analyses of PEDV E, M, and N genes available in GenBank. The tree was constructed using the neighbor-joining method (bootstrap n = 1000) based on MEGA7. a. E gene sequence. b. M gene sequence. c. N gene sequence.

Table S1 Continuous B-cell epitopes of CH/HLJ/18 S protein.

| Peptide | Start | End | Length |
| --- | --- | --- | --- |
| **CH/HLJ/18 protein model** | | | |
| **Bepipred 1.0** |  |  |  |
| PKNQGVN | 54 | 60 | 7 |
| KHPTAS | 68 | 73 | 6 |
| **Bepipred 2.0** |  |  |  |
| KNQGVNST | 55 | 62 | 8 |
| YCAGKHPTA | 64 | 72 | 9 |
| **BJ-2011-1 protein model** | | | |
| **Bepipred 1.0** |  |  |  |
| PIGENQGVN | 54 | 62 | 9 |
| QHPTASG | 70 | 76 | 7 |
| **Bepipred 2.0** |  |  |  |
| IGENQGVNST | 55 | 64 | 10 |
| WYCAGQHPTA | 65 | 74 | 10 |

Table S2 Discontinuous B-cell epitopes of CH/HLJ/18 S protein by ElliPro.

| Residues | Number of residues | Score |
| --- | --- | --- |
| **CH/HLJ/18 protein model** |  |  |
| N31, F32, R33, R34, F35, F36, S37, K38, F39, V41, Q42, A43, P44, A45, V46, Y52, L53, P54, K55, N56, Q57, G58, V59, N60, S61, T62, W63, Y64, C65, A66, G67, K68, H69, P70, T71, A72, S73, G74, V75, H76, I78, F79, V80, S81, H82, I83, R84, G85, G86, H87, G88, F89, Q95, E96, P97, A110, T111, N112, G113, N114, T115, N116, A154, H155, M156, S157, E158, H159, S160, V161, S188, T192, C194, Y195, N196, S197, G198, G199, C200, A201, M202, Q203, Y204, V205, Y206, E207, P208, T209, Y210, Y211, M212, L213, N214, V215, T216, S217, A218, G219, E220, D221, G222, I223, S224, Y225, Q226, P227, C228, T229, A230, N231, C232, N306, G307 | 113 | 0.668 |
| **BJ-2011-1 protein model** | | |
| N31, F32, R33, R34, F35, F36, S37, K38, F39, V41, Q42, A43, P44, A45, V46, Y52, L53, P54, I55, G56, E57, N58, Q59, G60, V61, N62, S63, T64, W65, Y66, C67, A68, G69, Q70, H71, P72, T73, A74, S75, G76, V77, H78, I80, F81, V82, S83, H84, I85, R86, G87, G88, H89, G90, F91, Q97, E98, P99, A112, T113, N114, G115, N116, T117, N118, A156, H157, M158, S159, E160, H161, S162, V163, S190, T194, C196, Y197, N198, S199, G200, G201, C202, A203, M204, Q205, Y206, V207, Y208, E209, P210, T211, Y212, Y213, M214, L215, N216, V217, T218, S219, A220, G221, E222, D223, G224, I225, S226, Y227, Q228, P229, C230, T231, A232, N233, C234, N308, G309 | 115 | 0.668 |

Table S3. Discontinuous B-cell epitopes of CH/HLJ/18 S protein by DiscoTope.

| Residue | Position | Contact number | Propensity score | Discotope score |
| --- | --- | --- | --- | --- |
| **CH/HLJ/18** |  |  |  |  |
| K | 55 | 13 | 0.663 | -5.837 |
| N | 56 | 12 | 1.424 | -4.576 |
| G | 58 | 15 | 0.35 | -7.15 |
| **BJ-2011-1** |  |  |  |  |
| I | 55 | 13 | -0.74 | -7.24 |
| G | 56 | 13 | -0.622 | -7.122 |
| Q | 59 | 14 | 0.744 | -6.256 |
