# Supplementary figures and images for: Porcine epidemic diarrhea virus strain CH/HLJ/18 isolated in China: characterization and phylogenetic analysis
Source: Virol J. 2024 Jan 24;21:28. doi: 10.1186/s12985-023-02233-6 (PMC10807084; doi:10.1186/s12985-023-02233-6)

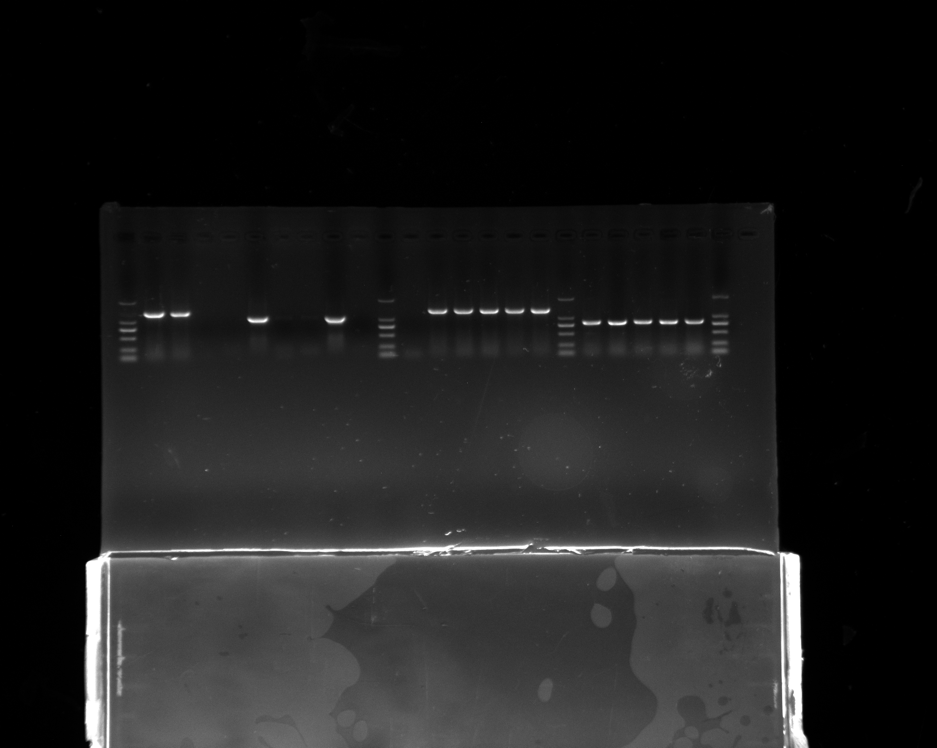


Figure 1a


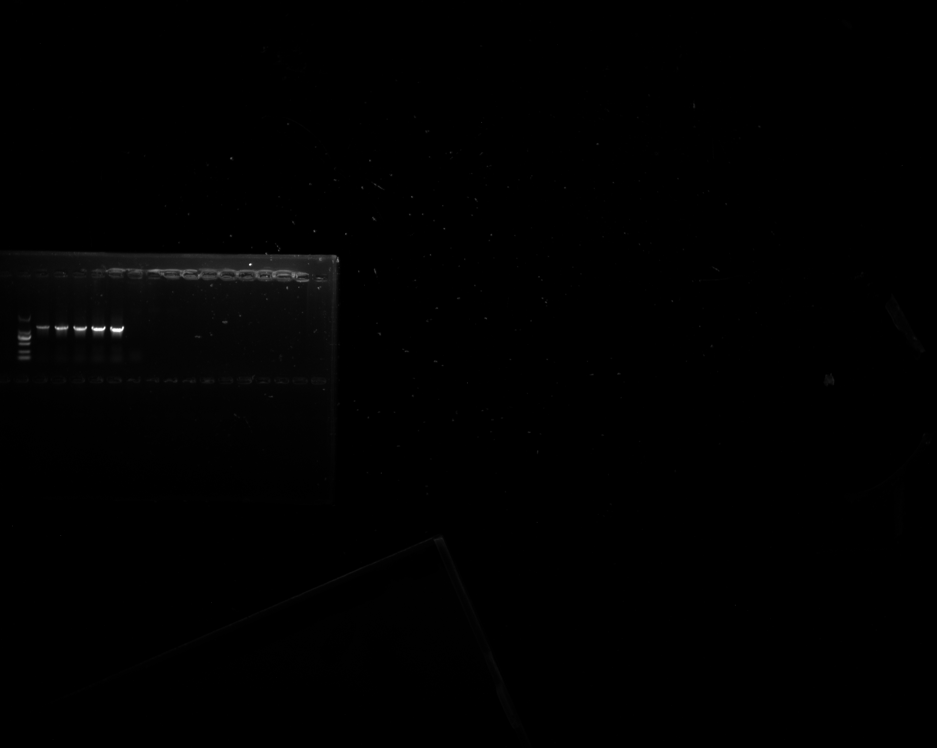


Figure 1c (Left)


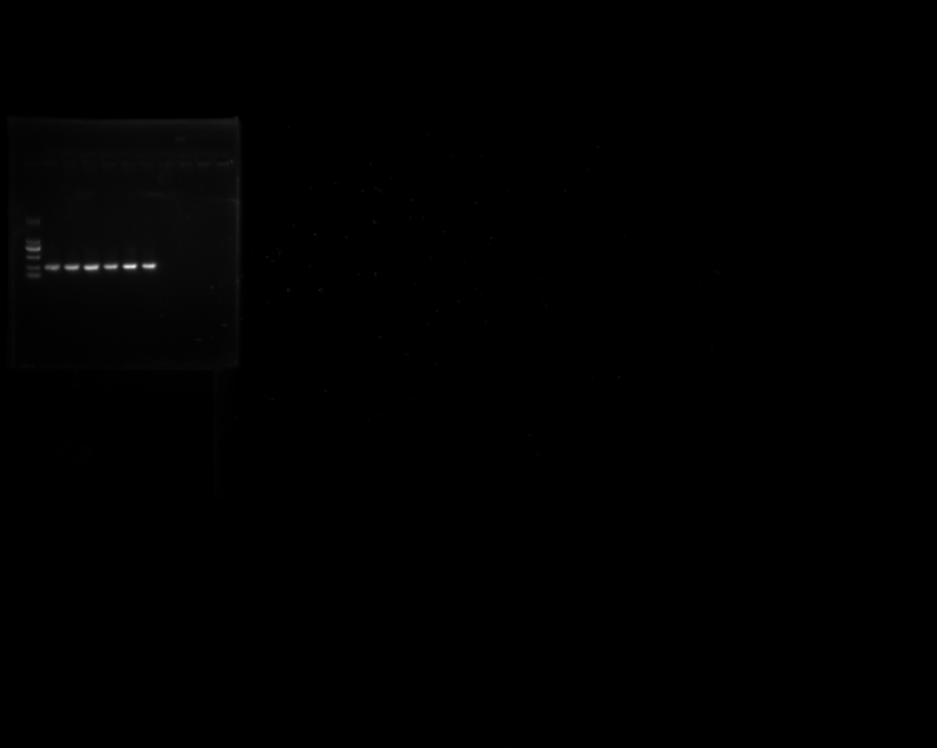


Figure 1c (Right)

Supplement: Supplementary file 2 — Supplementary Material 2 [file 12985_2023_2233_MOESM2_ESM.docx]
